# Apathy and White Matter Integrity in Alzheimer’s Disease: A Whole Brain Analysis with Tract-Based Spatial Statistics

**DOI:** 10.1371/journal.pone.0053493

**Published:** 2013-01-03

**Authors:** Changtae Hahn, Hyun-Kook Lim, Wang Yeon Won, Kook Jin Ahn, Won-Sang Jung, Chang Uk Lee

**Affiliations:** 1 Department of Psychiatry, Seoul Saint Mary’s Hospital, College of Medicine, The Catholic University of Korea, Seoul, Republic of Korea; 2 Department of Psychiatry, Saint Vincent’s Hospital, College of Medicine, The Catholic University of Korea, Suwon, Republic of Korea; 3 Department of Psychiatry, The Saint Paul Hospital, College of Medicine, The Catholic University of Korea, Seoul, Republic of Korea; 4 Department of Radiology, Seoul Saint Mary’s Hospital, College of Medicine, The Catholic University of Korea, Seoul, Republic of Korea; 5 Department of Radiology, Saint Vincent’s Hospital, College of Medicine, The Catholic University of Korea, Suwon, Republic of Korea; University of Manchester, United Kingdom

## Abstract

The aim of this study was to investigate the microstructural alterations of white matter (WM) in Alzheimer’s disease (AD) patients with apathy and to observe the relationships with the severity of apathy. Sixty drug-naïve subjects took part in this study (30 apathetic and 30 nonapathetic subjects with AD). The loss of integrity in WM was compared in AD patients with and without apathy through measurement of fractional anisotropy (FA) using by tract-based spatial statistics (TBSS). In addition, we explored the correlation pattern between FA values and the severity of apathy in AD patients with apathy. The apathy group had significantly reduced FA values (p_corrected_<0.05) in the genu of the corpus callosum compared to the nonapathy group. The severity of apathy was negatively correlated with FA values of the left anterior and posterior cingulum, right superior longitudinal fasciculus, splenium, body and genu of the corpus callosum and bilateral uncinate fasciculusin the apathy group (p_corrected_<0.05). This study was the first to explore FA values in whole brain WM in AD patients with apathy. The findings of these microstructural alterations of WM may be the key to the understanding of underlying neurobiological mechanism and clinical significances of apathy in AD.

## Introduction

Alzheimer’s disease (AD) remains one of the most debilitating illnesses worldwide. In addition to the serious cognitive decline including memory impairment, AD is accompanied by a number of neuropsychiatric symptoms which are also equally as important as memory decline in the clinical profile. Among the neuropsychiatric symptoms, apathy is known as the most frequent and serious behavioral symptom in AD. Since Marin presented apathy as a disorder of motivation [1], most of the contemporary definitions consider it as a lack of goal-directed behavior, cognition, or emotions [2], [3], [4], [5], [6]. The prevalence rate of apathy in AD is known to be in the range from 31% to 75% in AD patients [7], [8], [9]. Previous studies have reported that apathetic AD patients heavily rely on others despite having their own capability of performing activities and they have a higher rate of early institutionalization [10]. Additionally, apathetic AD patients are related to faster functional and cognitive decline [11].

To date, previous studies have tried to explore the neural substrates of apathy in AD. Although they suggested frontostriatal circuit dysfunction including the anterior cingulate, putamen and caudate nucleus [12], [13], the results were rather inconsistent. Moreover, the results were mainly concentrated on the gray matter change in AD patients with apathy. As evidenced by researches on white matter (WM), AD is known to be not only associated with regional gray matter (GM) change but also with disconnections between cortical brain regions [14]. Therefore, exploration of WM change might be as important as GM change in understanding the neurobiological mechanism of apathy in AD. Diffusion tensor imaging (DTI) can detect microstructual characteristics in the organization allowing for its implications in showing the connections between brain regions [15], [16], [17]. Several investigators have provided evidence of WM integrity alterations in AD as measured by fractional anisotropy (FA) derived from DTI [18], [19], [20].

To the best of our knowledge, there have been two DTI studies about WM FA values in AD patients with apathy. One study used the region of interest (ROI) approach and reported that AD patients with apathy showed decreased FA values in the left anterior cingulum and the FA values in the left anterior cingulum had a linear relationship with the severity of apathy [21]. The other study, using voxel-based morphometry (VBM)-style approach, reported negative correlations between the apathy scale and FA values in the right anterior cingulate, right thalamus, and bilateral parietal regions [22]. The ROI analyses were able to control for Type I error by limiting the number of statistical tests to few ROIs that were defined on the basis of information from previous studies [23]. However, ROI-based approaches were able to observe only specific areas and included the partial volume effect [24]. We conducted the whole brain analysis with an advanced method, tract-based spatial statistics (TBSS). Although VBM-style approaches can also analyze whole brain without prespecifying and prelocalising ROIs, a number of papers have discussed the limitations of VBM regarding imperfect image registration and random selection of smoothing factors [25]. TBSS was an improvement of the above-mentioned issues through projection into alignment-invariant tract representation, mean FA skeleton and no smoothing [25]. The aim of this study was to discover WM regions associated with apathy using the advanced method TBSS. We then examined the relationships between the significant changes of WM integrity and clinical variables among in AD patients with apathy.

## Materials and Methods

### Subjects

The study was conducted in accordance with the ethical and safety guidelines set forth by the local Institutional Review Board of the Catholic University of Korea and informed consent was obtained from all study subjects and their relatives. The local Institutional Review Board of the Catholic University of Korea approved this study (No. KC11RISI0900) and participants provided their written informed consents to participate in this study. Sixty right-handed and drug-naïve subjects took part in this study (30 apathetic and 30 nonapathetic subjects with AD). They were recruited from the Catholic Geriatric Brain MRI Database which was built through the outpatient psycho-geriatric clinic of St. Vincent’s Hospital located at the Suwon, South Korea from October 2009 to October 2011. All AD patients satisfied the following conditions: 1) fulfill the National Institute of Neurological and Communication Disorders and Stroke/Alzheimer Disease and Related Disorders Association (NINCDS-ADRDA) criteria for probable AD [26] and 2) have a score on the Clinical Dementia Rating Scale (CDR)≥1 [27]. We used a set of diagnostic criteria for apathy by Robert et al. [28] to separate the subjects into apathy and nonapathy groups. Participants who had other neurological or psychiatric conditions were excluded from the study. The severity of apathy was assessed with the apathy inventory (IA) [29]. Other neuropsychiatric conditions were also evaluated with a close psychiatric examination and the Neuropsychiatric Inventory (NPI) [30]. We only included participants with a 0 score in the depression/dysphoria domain of NPI to control for an important confounder, depressive symptoms.

### MRI Acquisition

All participants underwent MRI scans on a 3-Tesla whole body scanner equipped with an 8-channel phased-array head coil (Verio, Siemens, Erlangen, Germany) for the TBSS analysis. The scanning parameters of the DTI sequences were as follows: echo planar imaging, TR = 9300 ms, TE = 94 ms, field of view  = 192 mm, voxel dimension  = 2 mm isotropic, B-value  = 1000, gradients applied  = 20 isotropically, and distributed and acquisition time  = 21 min.

The evaluation of white matter hyperintensities (WMH) was provided by T2 weighted and Fluid Attenuated Inversion Recovery (FLAIR) images. All images were rated by a board-certified geriatric psychiatrist using the Fazekas scale [31]. All subjects scored 0 on the Fazekas scale.

### Diffusion Tensor Image Processing

For DTI data preprocessing, the FMRIB’s Diffusion Toolbox (FDT), which was a part of the FMRIB’s Software Library (FSL) program, was used. We visually inspected all the raw DTI data, and then corrected eddy current and head motion effect using FMRIB’s Linear Image Registration Tool (FLIRT) which was a part of FSL [32]. Brain images were extracted using the brain extraction tool (BET) [33] implemented in FSL. A diffusion tensor model was fitted at each voxel using the DTIfit program included in FSL. Thereafter, the fractional anisotropy (FA) map was calculated.

The voxel-wise statistical analysis was carried out by TBSS which was also a part of FSL. To explain in brief, we aligned each participant’s FA image onto a higher-resolution FA standard space (Montreal Neurological Institute [MNI] atlas) by the nonlinear registration method, FMRIB’s Nonlinear Image Registration Tool (FNIRT). The nonlinearly registered FA images were then averaged. The derived mean FA image was then minimized to create a template skeleton. The mean FA skeleton was further thresholded by an FA value of 0.2 to exclude the skeleton voxels which might lead to erroneous interpretations due to gray matter or cross-subject image misalignment. Finally FA data were projected onto the mean FA skeleton. TBSS analyses were carried out to compute for clusters showing a significant change between the AD subjects with apathy and AD subjects without apathy. Identification of WM areas of significant differences between two groups was based on the ICBM-DTI-81 white-matter and JHU white-matter tractography atlases (www.fmrib.ox.ac.uk/fsl/data/atlas-descriptions.html#wm).

### Statistical Analyses

Statistical analyses for demographic data were performed with the Statistical Package for Social Sciences software–SPSS (version 12.0, Chicago IL, USA). For assessing the potential differences between the apathy group and nonapathy group, the independent t test and the χ2 test were used. All statistical analyses had a two-tailed α level of <0.05 for statistical significance.

General linear model (GLM) was applied across all subjects to identify the brain regions in which the apathy group showed significant differences in FA relative to the nonapathy group. The WM regions showing correlations with IA scores were also examined. We performed correlation analyses to study the relationship between IA scores and FA values using IA scores as regressor in the framework of a GLM. The effects of age, education, gender and total intracranial volume (TIV) were regressed out in these models. A permutation-based approach accounting for “family wise errors” was used to control for multiple comparisons [34]. Permutation-based inference cluster size (t>1, p<0.05) was used to test whether FA values were significantly reduced in the apathy group compared with the nonapathy group.

## Results

Table 1 shows the baseline demographic data for the two different groups. No significant difference was observed in age, education, gender, CDR or TIV between the apathy group and nonapathy group. Compared with the nonapathy group, the apathy group had higher scores in the apathy/indifference domain of NPI and IA (p<0.05).

**Table 1:** Demographic and clinical characteristics of the study participants.

	Apathy group (*N* = 30)	Nonapathy group (*N* = 30)	*P*-value
Age (years±SD)	75.5±5.4	77.4±7.8	NS
Education (years±SD)	3.0±2.7	3.1±4.3	NS
Sex (M:F)	15∶15	13∶17	NS
IA total score	18.5±4.3	5.0±4.4	<0.0001
CDR	1.4±0.5	1.2±0.4	NS
AINPI	7.2±1.0	3.4±3.0	<0.0001
DDNPI	0	0	NS
TIV, (mm^3^±SD)	1319831.8±134304.3	1321943.3±113304.4	NS

**Figure 1:**
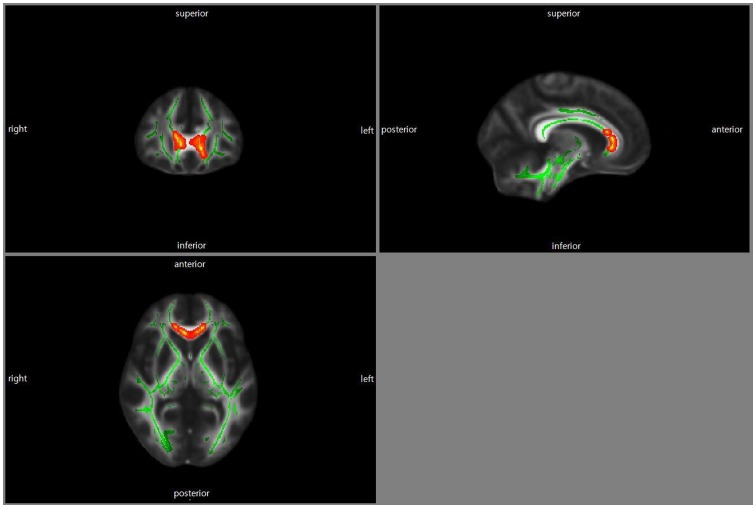
Comparison of the nonapathy group versus the apathy group. Statistical maps corrected for age, education, sex, and TIV showing lower FA values in AD patients with apathy relative to AD patients without apathy. The results were color-coded by p_corrected_<0.05. Red color denotes regions of declined FA values. AD, Alzheimer’s disease; CDR, Clinical Dementia Rating Scale; FA, Fractional Anisotropy; TIV, Total Intracranial Volume.

AINPI, Apathy/Indifference domain of Neuropsychiatric Inventory; CDR, Clinical Dementia Rating Scale; DDNPI, Depression/Dysphoria domain of Neuropsychiatric Inventory; IA, Apathy Inventory; TIV, total intracranial volume;

Compared to the nonapathy group, the apathy group had reduced FA values in the genu of the corpus callosum (Fig. 1, p_corrected_<0.05).

**Figure 2:**
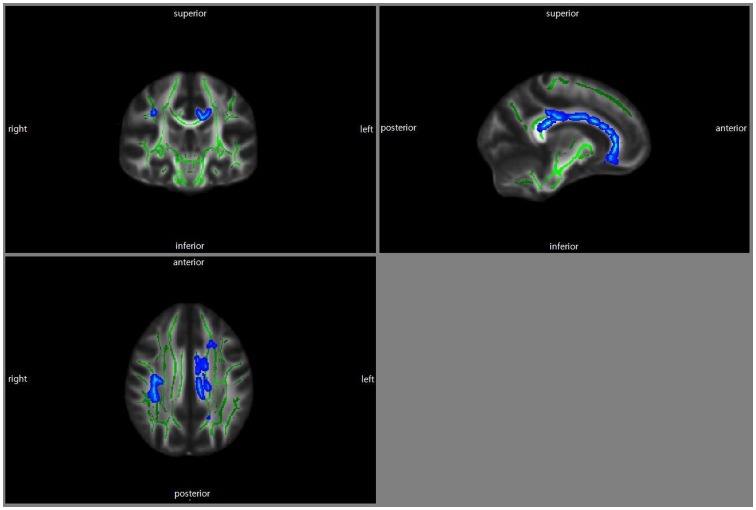
Correlation between IA scores and FA values. Statistical maps showing the regions of lower FA values correlated with IA scores. The results were color-coded by p_corrected_<0.05. FA, Fractional Anisotropy; IA, Apathy Inventory.

In the correlation analysis of apathetic subjects, IA scores were negatively correlated with the areas in the left anterior and posterior cingulum, right superior longitudinal fasciculus, splenium, body and genu of the corpus callosum and bilateral uncinate fasciculus (Fig. 2, p_corrected_<0.05). There was no significant correlation between IA scores and FA values in nonapathetic subjects.

## Discussion

Although the present study is not the first DTI analysis of AD patients with apathy, to the best of our knowledge, it is the first study to evaluate whole brain WM integrity changes in AD patients with apathy using unbiased and advanced method, TBSS. Because all the participants in the present study had a 0 score on the Fazekas scale and in the depression/dysphoria domain of NPI, we were able to ignore the effects of WMH and depression. A previous study by Starkstein et al. [35] reported that frontal WMH were closely associated with apathy in AD. In addition, the effects of medication on WM structure changes were not investigated because all participants were drug-naive. In fact, a previous DTI study showed that FA decline was preserved in the posterior body of the corpus callosum in the AD group with galantamine treatment compared to the placebo group [36].

Our findings showed that AD patients with apathy had lower FA values in the genu of the corpus callosum than those without apathy (Fig. 1). In addition, FA values of the genu, body and splenium of the corpus callosum were negatively correlated with the severity of apathy in the apathetic AD group (Fig. 2). The corpus callosum is a thick plate of fibers that reciprocally interconnects broad regions of the corresponding lobes of the cortex of the left and right side [37]. Also, the corpus callosum provides a link with subcortical structures, such as the basal ganglia [38]. According to previous studies, the anterior part of the corpus callosum contains interconnecting fibers from the prefrontal cortex [37] which are associated with the feeling of motivation. Therefore, our findings suggest that communication failure between brain structures might be associated with the apathetic symptoms of AD patients. Furthermore, the distinctive association pattern between the severity of apathy and the integrity of the corpus callosum might reflect slowed initiation and longer reaction times on tasks involving hemispheric transfer or integration between regions in apathetic AD patients [38]. Hoare et al. [39] showed a negative correlation between apathy severity and FA values in specific areas including the genu of the corpus callosum. Kang et al. [40] reported that apathy was related to the involvement of the corpus callosum in stroke patients.

In this study, we also observed that IA scores had an inverse linear relationship with FA values in the right superior longitudinal fasciculus and bilateral uncinate fasciculus in AD patients with apathy (Fig. 2). Our results are different from those of Kim el al. [21] who reported that the correlation between apathy severity and the uncinate fasciculus was not significant. Previous studies reported that the uncinate fasciculus as well as major components of the superior longitudinal fasciculus terminated at the prefrontal cortex [41], [42]. The prefrontal cortex is associated with several neuropsychiatric symptoms including motivation and attention. A previous study revealed that the uncinate fasciculus showed a significant relationship with apathy in amnestic mild cognitive impairment patients [43]. In addition, the present research showed that FA values of the superior longitudinal fasciculus were significantly associated with the severity of apathy. A previous study revealed a close relationship between the decline of FA in the superior longitudinal fasciculus and apathy in amnestic mild cognitive impairment patients [43]. Although there have only been few studies regarding the relationships between apathy and the superior longitudinal fasciculus, the superior longitudinal fasciculus is known to be involved in attentional network and working memory [44], [45]. Moreover, some investigators reported that apathy was associated with attention and working memory [46], [47]. In this context, we suggest that the superior longitudinal fasciculus might be associated with the severity of apathy in AD.

Our findings also showed that the severity of apathy negatively correlated with FA values in the left cingulum bundle, constituting (Fig. 2) the large bundle of fibers of the anterior and posterior cingulate gyrus [37]. The anterior cingulate cortex functions control the relationship between the emotional limbic system and autonomic portions of the nervous system [37] as well as mediating motivation. The posterior cingulate cortex is associated with memory, navigation, self-processing or consciousness [48]. Therefore, disconnections between the cingulate cortex and other brain structures via the cingulum might be closely related to apathy. Previous studies revealed a close relationship between the cingulated cortex and apathy [12], [49], [50], [51] and several studies reported the association between the decline of the cingulum FA and apathy [21], [43].

Apathy could be directly associated with neural substrates which are suggested in this study. Our findings could be interpreted as a result of an extension of frontostriatal circuit suggested by previous papers [3], [52]. However, the findings of this study might be the result of common tendency in AD with apathy rather than a direct reflection of neural substrates of apathy. A previous study by Douaud et al. [53] reported that WM microstrudtural changes in the cingulum, uncinate fasciculus, corpus callosum and superior longitudinal fasciculus were observed in patients with AD. Moreover, they showed that the significant difference between MCI and AD was essentially confined to the corpus callosum. Another study by Liu et al. [54] reported that neurofiber tracts with decreased FA were the parahipoocampal WM, cingulum, uncinate fascuculus, inferior and superior longitudinal fasciculus, corpus callosum, fornix, tracts in the brain stem, and cerebellar tracts. Indeed, neural substrates reflecting the progression from normal to pathological state in AD spectrum shared the regions associated with apathy from our findings. Apathy, at least in AD spectrum, could be a risk factor or a biomarker of disease progression. The clinical implications of apathy has been actively discussed already, and several papers suggested a faster cognitive decline in AD patients with apathy [11] and higher converting rates from MCI to AD in MCI patients with apathy [55]. Therefore, apathy of the clinician could form the basis of aggressive examination and treatment.

The neural substrates we suggested might be inconsistent in comparison to the results of previous studies about WM integrity of AD patients with apathy. The environment and the treatment of the patient would be key components in explaining apathy and other behavioral and psychological symptoms of dementia (BPSD). It might be one of the reasons for the previously contradictory results.

The current study has some limitations. First, sample size might not be large enough to detect every brain area associated with apathy. Second, patients with early AD were included in this study. We could obtain more accurate results by reducing potential confounders because early AD patients had a relatively smaller number of neuropsychiatric symptoms than those in advanced AD patients. However, it might be difficult to generalize about all stages of AD from our findings with early AD patients. Furthermore, all subjects had no depressive symptoms and no white matter lesions. This might be a rather selected and unusual group of AD patients, which could make our results less generalizable. Third, in order to confirm the clinical significance about disease progression we suggested, additional longitudinal studies regarding MCI and AD patients with apathy should be conducted.

### Conclusions

In this study, we showed that the genu of the corpus callosum had lost integrity in AD patients with apathy. In addition, we demonstrated significant correlations between the severity of apathy and loss of integrity of the left anterior cingulum, left posterior cingulum, right superior longitudinal fasciculus, splenium, body, and genu of the corpus callosum and bilateral uncinate fasciculus. These WM microstructural changes might be the key to the underlying neurobiological mechanism of apathy in AD.
